# Are the Assioma Favero Power Meter Pedals a Reliable Tool for Monitoring Cycling Power Output?

**DOI:** 10.3390/s21082789

**Published:** 2021-04-15

**Authors:** Víctor Rodríguez-Rielves, José Ramón Lillo-Beviá, Ángel Buendía-Romero, Alejandro Martínez-Cava, Alejandro Hernández-Belmonte, Javier Courel-Ibáñez, Jesús G. Pallarés

**Affiliations:** 1 Human Performance and Sports Science Laboratory, University of Murcia, 30100 Murcia, Spain; Victor.RRielves@uclm.es (V.R.-R.); jr.lillo@ua.es (J.R.L.-B.); Angel.buendiar@um.es (Á.B.-R.); Alejandro.martinez12@um.es (A.M.-C.); alejandro.hernandez7@um.es (A.H.-B.); courel@um.es (J.C.-I.); 2 Exercise Physiology Laboratory, University of Castilla-La Mancha, 13001 Toledo, Spain

**Keywords:** cycling, mobile power meter, testing, load monitoring

## Abstract

This study aimed to examine the validity and reliability of the recently developed Assioma Favero pedals under laboratory cycling conditions. In total, 12 well-trained male cyclists and triathletes (VO_2max_ = 65.7 ± 8.7 mL·kg^−1^·min^−1^) completed five cycling tests including graded exercises tests (GXT) at different cadences (70–100 revolutions per minute, rpm), workloads (100–650 Watts, W), pedaling positions (seated and standing), vibration stress (20–40 Hz), and an 8-s maximal sprint. Tests were completed using a calibrated direct drive indoor trainer for the standing, seated, and vibration GXTs, and a friction belt cycle ergometer for the high-workload step protocol. Power output (PO) and cadence were collected from three different brand, new pedal units against the gold-standard SRM crankset. The three units of the Assioma Favero exhibited very high within-test reliability and an extremely high agreement between 100 and 250 W, compared to the gold standard (Standard Error of Measurement, SEM from 2.3–6.4 W). Greater PO produced a significant underestimating trend (*p* < 0.05, Effect size, ES ≥ 0.22), with pedals showing systematically lower PO than SRM (1–3%) but producing low bias for all GXT tests and conditions (1.5–7.4 W). Furthermore, vibrations ≥ 30 Hz significantly increased the differences up to 4% (*p* < 0.05, ES ≥ 0.24), whereas peak and mean PO differed importantly between devices during the sprints (*p* < 0.03, ES ≥ 0.39). These results demonstrate that the Assioma Favero power meter pedals provide trustworthy PO readings from 100 to 650 W, in either seated or standing positions, with vibrations between 20 and 40 Hz at cadences of 70, 85, and 100 rpm, or even at a free chosen cadence.

## 1. Introduction

The use of power meters in cycling has been on the rise in recent years, making accessible, valuable information for training, that was only available with impractical and expensive ergometers [1,2]. Portable power meter devices overcome important drawbacks of laboratory testing, allowing the use of cyclists’ own bicycles, so that decisive metrics such as the crank width (Q–factor), crank length, and geometry-related variables are replicated in the test [3]. Commercial indoor stationary cycle training, cycling treadmills, or rollers are a valid and reliable alternative to recreate outdoor cycling conditions, both for testing [4,5,6] and training [7]. While these tools simulate outdoor cycling, they do not allow recording during real outdoor environments (e.g., missing air drag and downhill sections or increasing dehydration), which may alter the metrics [8,9] and limit to apply the results to real-life situations.

The development of wearable power meters with micro-sensors attached to the bicycle crank, pedals or wheel, constitutes a milestone for cycling, giving rise to the creation of new devices, which can track cyclists’ performance in real settings. The first approach was the SRM (professional model; Schoberer Rad Messtechnik, Julich, Germany) crankset (strain gauges), which remains as the Gold-Standard to measure the bicycle power output (PO) outside the laboratory [10,11,12]. Since then, emerging alternatives have been demonstrated to be valid and reliable, such as the wheels Powertap Hub [13,14,15] or the pedals Garmin Vector [1,15,16,17,18] and Powertap P1 [19,20,21]. In particular, due to their quick installation and use [1,15,16,17,18,19,20,21], the pedal power meters would represent a high practical technology to be used interchangeably in different bicycles (e.g., track, road, and time trials). Additionally, pedals are likely to reduce the loss of PO due to mechanical connections [12]. Recently, a new brand of pedal power meters called Assioma Favero (Favero Electronics SRL, Arcade TV, Italy) has been launched on the market. In addition to reduced weight and size, the lower of this device compared to the traditional SRM makes the PO measurement more affordable for practitioners. Nevertheless, there is scarce information about the measurement errors of this commercially available technology.

In practice, the main goal of tracking PO is to quantify the real effort incurred during training or competition, and also to determine changes in performance throughout the season [22]. For this purpose, it is essential to determine the measurement error of the device in use to guarantee that these errors are narrow enough to determine the true PO achieved by the cyclists [23,24]. Accordingly, if the error exceeds the expected changes, the device renders it completely useless for its intended purpose [25]. Hence, to be sure of the certainty of the outcomes, emerging power meter devices should be repeatedly tested across a variety of cycling conditions to determine how well they respond to changes in the cadence, the pedaling position (seated or stand), the PO, or the vibration [15].

Therefore, considering the practical advantages that the pedals power meter would provide to the PO prescription and monitoring, as well as the need to comprehensively analyze the suitability of this type of technologies to be used on the daily basis, this study aimed to examine the validity and reliability of the recently developed Assioma Favero pedals under laboratory cycling conditions.

## 2. Materials and Methods

### 2.1. Experimental Design

This study followed a repeated measures design to determine the validity and test–retest reliability of three units of the new power meter pedals Assioma Favero against the gold-standard SRM crankset. After a familiarization session, each participant completed the following cycling tests: three counterbalanced, graded exercises tests (GXT) at different cadences (70, 85, 100 revolutions per minute, rpm) and sub-maximal workloads (100, 150, 200, 250, 300, 350 Watts, W) in a seated position, three GXT at four sub-maximal workloads (free cadence; 250, 350, 450, 550 W) in a standing position, and a ramp vibration protocol (from 20 to 40 Hz) at constant workload (200 W; 85 rpm). Finally, all cyclists performed a high-workload step protocol (450, 550, 650 W, in seated position, 85 rpm), as well as an 8-s maximal sprint test.

### 2.2. Subjects

A total of 12 well-trained male cyclists and triathletes volunteered to take part in this study. (M ± SD: age 27.9 ± 9.5 years; height 180.0 ± 7.8 cm; body mass 78.0 ± 16.4 kg; VO_2max_ = 65.7 ± 8.7 mL·kg^−1^·min^−1^ [26]). All subjects had more than 5 years of cycling training experience and followed a training routine of 6 h per week during the 12 months preceding the study. Athletes were all older than 18 years, were informed of the experimental procedures, and signed a written informed consent agreeing to participate in the study. Participants were asked to avoid strenuous exercise, caffeine, or alcohol for at least 24 h before each testing session. The study was conducted according to the Declaration of Helsinki, and was approved by the Bioethics Commission of Local University.

### 2.3. Testing Procedures

All tests were performed in the same facilities under standardized conditions (23.8 ± 2.4 °C; 39 ± 5% humidity). For the seated and standing GXTs, as well as the vibration tests, the SRM 172.5 mm crank power meter was fixed on a medium-size road bicycle (2010 Giant Giant-Bicycles, Taiwan; Aluminum alloy frame with carbon fiber fork). The rear wheel of the bicycle was removed and attached to a calibrated Cycleops Hammer [6] device with 10 speed (11–25 tooth) rear gear ratio and 39 to 53 tooth front gear ratio. For all tests, the gear ratio 53 × 15 was selected, and cyclists were not allowed to change it to prevent a potential effect of this variable on pedaling technique. The zero–offset of the Assioma Favero power meter pedals was set before each testing session. For the vibration tests, the whole system (Bike trainer and bicycle) was installed over a vibrating plate (Merit Fitness V2000) with the front fork of the bicycle attached to a Kickr Climb Indoor Grade Simulator (Wahoo Fitness, Atlanta, GA, USA) for stability and to compensate the height of the vibration platform (0% slope). The bicycle seat height position was matched to the cyclist’s training geometry. For the high-workload step protocol (GXT ≥ 450 W) and the 8-s maximal sprint, the SRM crankset unit was installed in a friction belt cycle ergometer (Monark 847E Varberg, Sweden) to achieve the required mechanical resistance. The saddle and handlebar positions of the cycle ergometer were also matched to the cyclist’s training geometry. Data were transmitted to display units (Garmin 520, Garmin International Inc., Olathe, KS, USA) fixed on the handlebars. Calibration and set-up were conducted according to the manufacturer’s recommendations. Cyclists used their cycling shoes fitted with Look cleats.

### 2.4. Cyclings Tests

**Figure 1:**
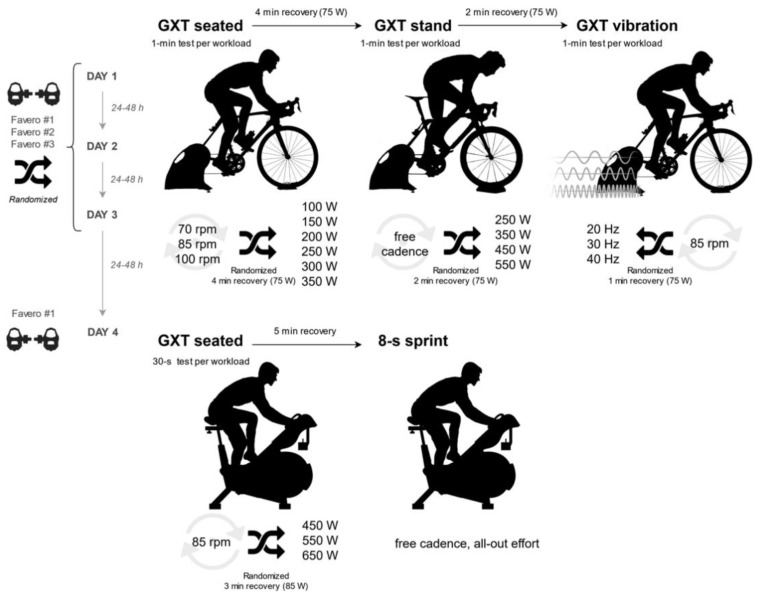
Experimental design including the five cycling tests.

Subjects visited the laboratory on four separate occasions to test the three Assioma Favero power meter pedals. All tests began with a standardized warm-up of 5 min at 75 W with a free chosen cadence and the Hammer set in the hyperbolic mode. Thereafter, subjects performed three randomized and counterbalanced 1-min GXT in seated position, one for each selected fixed cadence (70, 85, and 100 rpm), at six sub-maximal workloads (i.e., 100, 150, 200, 250, 300, and 350 W), separated by 4 min of recovery at 75 W with free chosen cadence [6] (Figure 1). The order of the three cadence levels was randomized to ensure that results were not altered due to increments on the ergometer break temperature or by the cyclists’ fatigue. After recovery, cyclists performed three 1-min GXT in standing pedaling position at 250, 350, 450 W, and 550 W with free chosen cadence. After 2 min of recovery at 75 W, subjects performed a vibration test, simulating common vibrations in road cycling [27]. The test consisted of a 1 min ramp exercise, bouts on a vibrating plate by steps of 10 Hz, increasing from 20 to 40 Hz, at 200 W with a pedaling cadence of 85 rpm. This complete protocol was repeated on three different occasions in a randomized and counterbalanced way, one for each Assioma Favero pedal units (Figure 1). In the fourth visit to the laboratory, subjects performed a 30-s, seated position, high-load GXT at 85 rpm in a friction belt cycle ergometer, with the resistances required to produce 450 W (5.3 kp), 550 W (6.4 kp), and 650 W (7.6 kp). Each step was followed by 3 min of recovery with 1 kp (85 W). Following a further 5-min recovery period, subjects were required to complete an 8-s maximal sprint test (verbally encouraged, all-out effort) starting from a complete stop with the pedal of the dominant leg placed at 90° from the vertical and against the resistance of 7.5% of the subject’s body mass (body mass × 0.075 kg) [28]. The four sessions were conducted at the same time of the day (10:00–13:30 h), and under similar environmental conditions (21–22 °C and 53–62% humidity) [29].

### 2.5. Data Collection

Records for PO (W) and cadence (rpm) were collected at 1 Hz using a Garmin 520 cycling computer for the Assioma Favero pedals and the Power Control VIII (professional model, Schoberer Rad Messtechnik, Julich, Germany) for the SRM crankset. Data for GXT and vibration tests included the 15th to the 55th s of each 60 s steps, to allow the ergometer enough time to stabilize the assigned breaking load [12]. Similarly, data from the 8th and the 28th s of each 30 s steps were considered for the high-load GXT tests, while peak PO and the mean PO for the first 6 s of the sprints were included. Data were exported and analyzed using the publicly available software (Golden Cheetah, version 3.5) and Microsoft Excel 2016. 

### 2.6. Statistical Analysis

Standard statistical methods were used for the calculation of means, standard deviations (SD), coefficient of variation (CV), and standard error of measurement (SEM) [30]. Intraclass correlation coefficients (ICC) were used to determine the relationship between the power outcomes of the SRM and the Assioma Favero pedals. Bland–Altman plots were used to examine heteroscedasticity and assess the systematic errors and their 95% limits of agreement (LoA = bias ± 1.96 SD) [31]. Levels of acceptable disagreement were proposed at ≤2% to identify true changes in performance after a training intervention [24]. Homoscedasticity was confirmed by Levene’s test. Repeated-measures ANOVA was conducted to determine the statistical effects of the different devices in the PO metrics across the different GXT tests. Partial eta squared was calculated to estimate the effect size (ES), interpreted as small (0.02), medium (0.13), and large (0.26) [32]. Statistical significance was set as *p* ≤ 0.05. Analyses were performed using GraphPad Prism 6.0 (GraphPad Software, Inc., San Diego, CA, USA), SPSS software version 19.0 (SPSS, Chicago, IL, USA), and Microsoft Excel 2016 (Microsoft Corp, Redmond, WA, USA).

## 3. Results

**Figure 2:**
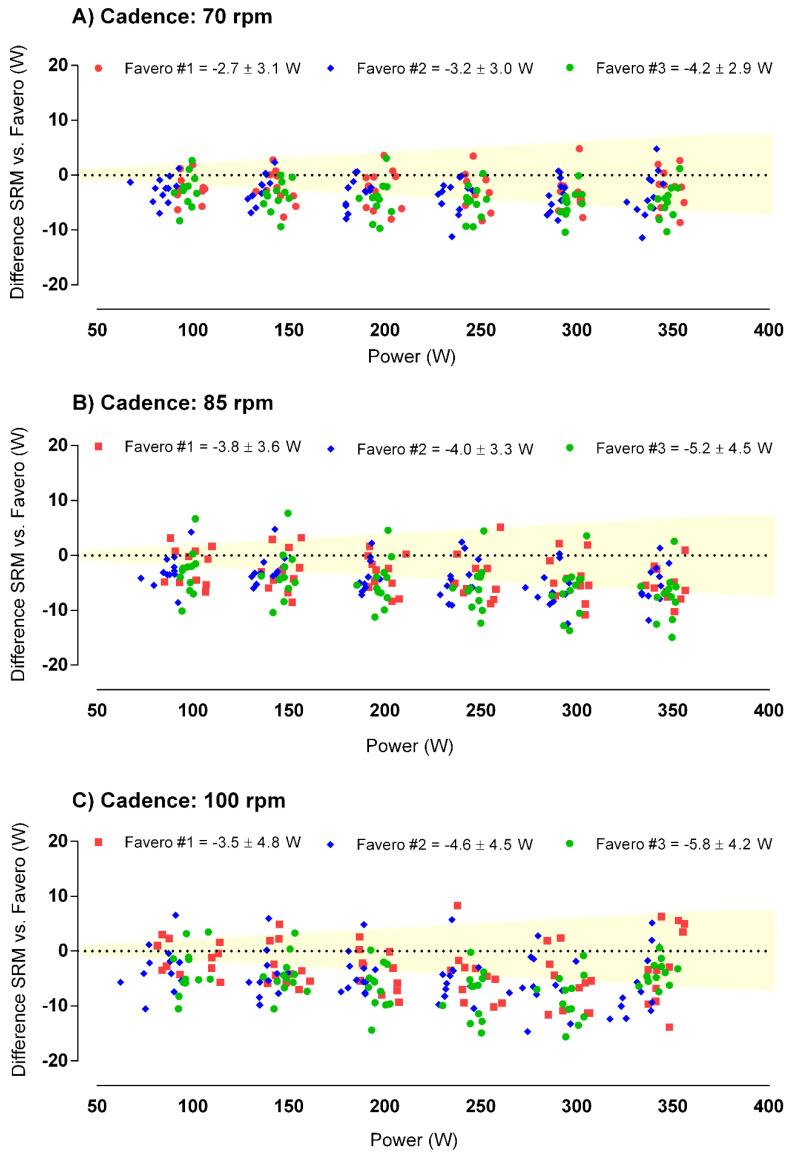
Bland–Altman plots showing the level of agreement between the three Favero Assioma pedals (markers) and the gold standard SRM crankset, during the seated graded exercises tests (GXT). Area shaded in yellow indicates an acceptable level of agreement ≤ 2% [24].

The three Favero Assioma pedals exhibited very high reliability during the tests (CV from 1.5 to 13.8%) comparable to the SRM (CV differences < 2%), and high ICC (from 0.741 to 0.999). SRM crankset and the three Favero Assioma showed similar PO in most conditions (Table 1), with extremely high agreement when pedaling between 100 and 250 W (SEM from 2.3 to 6.4 W). Greater PO produced a significant underestimating trend, especially in GXT seated at 300 W/70 rpm, GXT seated at 350 W/80 rpm, and GXT standing > 450 W (*p* < 0.05, ES > 0.22), with Favero showing from 1 to 3% lower PO than SRM consistently. In turn, all devices showed similar PO during [30], the GXT seated ≥ 450 W in the Monark. Vibrations ≥ 20 Hz significantly increased the differences up to 4% (*p* < 0.05, ES > 0.24). Peak and mean PO differed importantly between devices during the sprints (*p* < 0.03, ES > 0.39). Bland–Altman plots (Figure 2) confirmed that Favero Assioma pedals showed systematically lower PO than SRM, but produced low bias (1.5 and 7.4 W) and SD (4.7 and 10.0 W) for all testing conditions.

## 4. Discussion

The results of this study indicate that the Assioma Favero Pedals are a highly suitable tool for monitoring cycling performance in a wide range of workloads (100 to 650 W) and cadences (70, 85, and 100 rpm), different pedaling positions (seated and standing), and under vibration stress (20, 30, and 40 Hz). Importantly, the pedals slightly underestimated the PO compared with SRM readings, but errors are low enough to be handled in practice. To the best of our knowledge, this is the first study examining the validity and reliability of the recently commercialized Assioma Favero pedals. Stemming from this comprehensive research, coaches and researchers may be confident in using these portable power meters for cycling training and testing and benefit from their practical advantages. 

The SRM crankset constitutes the best alternative available to laboratory cycle ergometers, with extremely low variability (<1.0% for a 20-strain-gauge model, and <2.0% for the 4-strain-gauge model) [12]. According to our findings, the Assioma Favero readings were very similar to the SRM across the variety of conditions examined, considering a systematic underestimation of PO readings (from −2.7 ± 5.8 W to −6.0 ± 9.9 W), probably due to the strain gauges’ sensitivity or the signal processing [15]. These disparities are comparable to previously validated devices such as the Powertap P1 pedals (from −2.4 ± 4.8 W to −9.0 ± 5.3 W) [19], Garmin Vector Pedals (0.6 ± 6.2 W, 11.6 to 12.7 W; −11.6 to 12.7 W, −3.7 to 9.5 W) [1,15], Powertap Hub (2.9 ± 3.3 W; −3.7 to 9.5 W) [13], and Look Keo Power Pedal (4.6 ± 0.4 W; −15.9 to 13.9 W) [33]. Our results suggest that Assioma Favero pedals are therefore not only useful but also reliable for cycling load monitoring. In addition to the lower price in comparison with the SRM technology (>1.500 US), these pedal power meters have key advantages such as maintaining the usual riding position, the wheelset, and the crankset, as well as the reduced extra weight (microsensors attached to the pedals). Moreover, from a practical view, the ease installation of the Assioma Favero pedals allows athletes to use them interchangeably in different bicycles (e.g., track, road, and time trial). On the other hand, in comparison with other brands of pedal power meters, the features of the Assioma Favero pedals (cost ~800 US; weight ~151.5 g) make them a more affordable technology than the Garmin Vector (cost ~1400 US; weight ~156 g), as well as a lighter option than the Powertap P1 (cost ~750 US; weight ~194.5 g). 

An important contribution of the present study is that we examined a large variety of testing conditions, allowing us to conclude the effects of three big cycling concerns: pedaling positions, vibration, and extremely high loads. Whereas previous studies have included some of these conditions [1,15,16,17,21,34], this is the first time they have all been examined in the same experiment. Of interest, there was no substantial difference in the readings between standing and seated pedaling positions, even though it is known that standing pedaling causes lateral sways and affects the biomechanics of pedaling [35]. Furthermore, testing the device performance under vibration stress is quite important considering that 88% of the excitation power during a ride on the granular rough road falls within a 10–50 Hz frequency bandwidth [27]. Our results showed that Assioma Favero pedals had similar CV, bias, and SD of bias than SRM under vibration conditions, including high ICC values. However, readings could be altered ~4% by vibrations > 20 Hz.

The fact that the Assioma Favero pedals produce errors of ~2% compared to the SRM suggests that they are sufficiently accurate to track performance changes over time [24]. This result is similar to those observed in the Powertap Hub (1.7 to 2.7%) [13] and better than the ones found in the Garmin 3.1% [1] and Vector pedals (8.5 ± 4.0%) [17]. Despite the practical advantages they offer, the Assioma Favero Pedals are limited concerning their calibration. Static calibration is not possible because the pedals need a reading of the cadence [36]. Thus, the slope of the power curve cannot be adjusted, meaning that they will always be limited by the factory calibration. Accordingly, the pedal measurement should be checked regularly against a calibrated scientific SRM crankset. Given that the current experiment was conducted under laboratory settings, future research should address the reliability of the Assioma Favero Pedals in field conditions [15].

## 5. Conclusions

This study confirms that the new Assioma Favero pedals are valid and reliable mobile power meters to measure PO in cyclists. This portable power meter provides an alternative to more expensive laboratory ergometers while allowing cyclists to use their bicycles for testing, training, or competition purposes. The results demonstrate that the Assioma Favero power meter pedals provide trustworthy PO readings from 100 to 650 W, in either seated or standing positions, with vibrations between 20 and 40 Hz at cadences of 70, 85, and 100 rpm, or even at a free chosen cadence. Of note, pedals consistently underestimated the SRM readings by up to 4%, with differences depending on the cycling condition. 

## Figures and Tables

**Table 1:** Power outcomes for SRM crack set and the three Favero Assioma pedals.

	Mean (SD)		SEM	Mean (SD)		SEM	Mean (SD)		SEM	Within-Device Effect	
	SRM	Favero #1		SRM	Favero #2		SRM	Favero #3		*p*-Value	ES
**GXT seated [70 rpm]**											
100 W	100 (6)	97 (6)	2.3	100 (8)	97 (8)	2.8	98 (3)	96 (4)	2.7	0.399	0.078
150 W	250 (6)	143 (5)	2.5	250 (6)	145 (8)	2.6	250 (4)	142 (5)	3.2	0.132	0.165
200 W	200 (7)	197 (7)	2.9	200 (5)	197 (6)	3.1	199 (4)	194 (5)	3.8	0.165	0.155
250 W	249 (6)	246 (5)	3.1	250 (6)	246 (6)	3.6	249 (4)	244 (4)	3.9	0.1	0.186
300 W	300 (5)	296 (5)	3.3	300 (3)	296 (4)	3.3	299 (3)	294 (5)	4.0	0.046 *	0.269
350 W	350 (6)	348 (5)	3.1	350 (5)	346 (7)	3.8	349 (4)	344 (5)	4.0	0.071	0.209
**GXT seated [85 rpm]**											
100 W	100 (9)	98 (8)	2.8	100 (7)	97 (8)	3.1	99 (3)	96 (4)	3.5	0.454	0.066
150 W	149 (7)	146 (7)	3.3	149 (5)	147 (7)	2.6	148 (5)	145 (6)	3.9	0.377	0.085
200 W	201 (7)	197 (6)	3.2	200 (3)	196 (4)	3.4	200 (4)	195 (6)	4.6	0.099	0.2
250 W	250 (9)	246 (9)	3.9	250 (6)	246 (8)	4.0	250 (5)	244 (5)	5.1	0.152	0.162
300 W	300 (8)	296 (7)	4.1	299 (7)	294 (7)	4.1	300 (4)	294 (6)	5.5	0.109	0.186
350 W	350 (7)	345 (7)	4.3	350 (4)	345 (6)	4.4	350 (6)	343 (6)	6.1	0.035 *	0.275
**GXT seated [100 rpm]**											
100 W	100 (14)	98 (14)	2.1	100 (11)	97 (12)	4.2	100 (6)	96 (7)	3.8	0.647	0.034
150 W	150 (8)	147 (6)	3.3	149 (6)	145 (8)	5.1	151 (6)	146 (7)	4.0	0.153	0.153
200 W	199 (10)	195 (8)	3.7	200 (6)	195 (7)	5.2	199 (4)	193 (4)	5.3	0.08	0.202
250 W	249 (11)	245 (8)	4.7	250 (8)	245 (7)	4.6	250 (6)	242 (6)	6.4	0.08	0.202
300 W	300 (11)	293 (9)	5.5	300 (12)	294 (11)	5.6	300 (7)	292 (7)	6.7	0.102	0.18
350 W	349 (14)	343 (12)	5.3	350 (11)	342 (11)	6.5	350 (5)	340 (6)	7.4	0.124	0.178
**GXT stand [free cadence]**											
250 W	250 (9)	251 (7)	2.1	250 (9)	250 (9)	1.4	249 (8)	244 (7)	4.3	0.352	0.091
350 W	350 (7)	350 (6)	1.9	350 (8)	350 (9)	1.7	350 (8)	343 (9)	5.7	0.15	0.156
450 W	451 (10)	452 (12)	4.2	450 (7)	452 (9)	2.8	449 (10)	442 (10)	6.3	0.050 *	0.221
550 W	551 (14)	554 (16)	4.1	550 (10)	554 (13)	5.0	542 (28)	537 (24)	9.2	0.045 *	0.235
**GXT vibration [85 rpm]**											
20 Hz	200 (6)	196 (5)	4.4	200 (6)	195 (9)	4.4	201 (7)	193 (8)	5.7	0.106	0.186
30 Hz	200 (7)	196 (8)	3.8	200 (7)	193 (7)	5.8	201 (7)	193 (9)	5.9	0.043 *	0.244
40 Hz	200 (8)	194 (7)	5.0	200 (5)	194 (8)	5.3	201 (6)	192 (8)	6.3	0.024 *	0.272
**GXT seated [85 rpm]**											
450 W	449 (6)	449 (8)	3.5	—	—		—	—		0.708	0.013
550 W	544 (7)	545 (6)	3.0	—	—		—	—		0.671	0.017
650 W	645 (11)	647 (11)	3.4	—	—		—	—		0.306	0.095
**6-s sprints**											
Peak PO	1268 (278)	1156 (171)	127.5	—	—		—	—		0.023 *	0.386
Mean PO	1082 (181)	921 (119)	130.5	—	—		—	—		<0.001 *	0.758

SEM: Standard error of measurement. GXT: graded exercises tests, rpm: revolutions per minute. ES: Effect size. * Significant differences compared to the SRM device (*p* < 0.05).

## Data Availability

The data presented in this study are available on request from the corresponding author. The data are not publicly available due to privacy.
